# Translational reprogramming of colorectal cancer cells induced by 5-fluorouracil through a miRNA-dependent mechanism

**DOI:** 10.18632/oncotarget.17597

**Published:** 2017-05-03

**Authors:** Zeina Bash-Imam, Gabriel Thérizols, Anne Vincent, Florian Lafôrets, Micaela Polay Espinoza, Nathalie Pion, Françoise Macari, Julie Pannequin, Alexandre David, Jean-Christophe Saurin, Hichem C. Mertani, Julien Textoris, Didier Auboeuf, Frédéric Catez, Nicole Dalla Venezia, Martin Dutertre, Virginie Marcel, Jean-Jacques Diaz

**Affiliations:** ^1^ Univ Lyon, Université Claude Bernard Lyon 1, INSERM 1052, CNRS 5286, Centre Léon Bérard, Centre de recherche en cancérologie de Lyon, F-69373 Lyon, France; ^2^ Institut Curie, CNRS UMR 3348, Centre Universitaire, F-91405 Orsay, France; ^3^ EA7426, Université Lyon 1, Hospices Civils de Lyon, bioMérieux S.A. Pathophysiology of injury-induced immunosuppression (PI3), F69003 Lyon, France; ^4^ IGF, CNRS, INSERM, Université Montpellier, F-34094 Montpellier, France

**Keywords:** 5-fluorouracil, translation, translatome profiling, miRNA, colorectal cancer

## Abstract

5-Fluorouracil (5-FU) is a widely used chemotherapeutic drug in colorectal cancer. Previous studies showed that 5-FU modulates RNA metabolism and mRNA expression. In addition, it has been reported that 5-FU incorporates into the RNAs constituting the translational machinery and that 5-FU affects the amount of some mRNAs associated with ribosomes. However, the impact of 5-FU on translational regulation remains unclear. Using translatome profiling, we report that a clinically relevant dose of 5-FU induces a translational reprogramming in colorectal cancer cell lines. Comparison of mRNA distribution between polysomal and non-polysomal fractions in response to 5-FU treatment using microarray quantification identified 313 genes whose translation was selectively regulated. These regulations were mostly stimulatory (91%). Among these genes, we showed that 5-FU increases the mRNA translation of *HIVEP2*, which encodes a transcription factor whose translation in normal condition is known to be inhibited by mir-155. In response to 5-FU, the expression of mir-155 decreases thus stimulating the translation of *HIVEP2* mRNA. Interestingly, the 5-FU-induced increase in specific mRNA translation was associated with reduction of global protein synthesis. Altogether, these findings indicate that 5-FU promotes a translational reprogramming leading to the increased translation of a subset of mRNAs that involves at least for some of them, miRNA-dependent mechanisms. This study supports a still poorly evaluated role of translational control in drug response.

## INTRODUCTION

Translational control regulating one of the last steps of gene expression, plays a key role in tumor development [1, 2]. By finely regulating the synthesis of a specific subset of proteins, translational control contributes to tumor initiation, invasion and metastasis. In contrast, the role of translational control in anti-cancer drug response is just starting to emerge and large-scale analysis of the translatome has been carried out for only a few anti-cancer drugs [3–6].

Among the drugs commonly used in chemotherapy, 5-Fluorouracil (5-FU) is an anti-metabolite widely given in first-line of treatment in many types of solid cancers. For a long time, the 5-FU-induced cytotoxic effects were thought to result exclusively from its impact on DNA metabolism, in particular from 5-FU-induced inhibition of DNA synthesis and induction of DNA damage [7]. However, several evidences indicate that the cytotoxic effect of 5-FU also results from its capacity to alter RNA metabolism and mRNA expression [7]. 5-FU can be incorporated into all species of RNAs after its conversion into fluorouridine triphosphate (FUTP) and media complementation with uridine, which allowed recovery of normal RNA metabolism, compensate most of the 5-FU-induced cytotoxic effects [8–11]. Furthermore, exposure to 5-FU promotes a profound transcriptional reprogramming leading to modification of mRNA and miRNAs expression profiles that contributes in modifying cell fate [12–14].

Although 5-FU directly alters RNA metabolism and mRNA expression, the impact of 5-FU on translation has yet been poorly characterized. Several evidences indicate that 5-FU could alter translation. It has been shown that 5-FU affects processing and functions of two components of the translational machinery, ribosomal RNA (rRNA) and transfer RNA (tRNA) [15–18]. Moreover, genome-wide screening in yeast revealed that rRNA and tRNA processing factors mediate a part of 5-FU cytotoxicity [19–22]. Finally, two large-scale studies showed that 5-FU could regulate the translational output of a set of mRNAs [23, 24]. For some of these mRNAs, their global expression levels were not strongly affected by 5-FU, while their abundance in the polysomal fractions (i.e. ribosome-bound mRNAs) was modulated. Although these data raised the possibility that their translational efficiency might be regulated by 5-FU, it was not clearly addressed in these studies. Therefore, the regulation of translational efficiency by 5-FU has not been determined on a large-scale, and the molecular mechanisms mediating such regulations were not determined.

In the present study, to investigate whether treatment with a clinically relevant dose of 5-FU affects the translational efficiency of specific mRNAs, we performed a genome-wide analysis of the translatome by comparing the distribution of mRNAs within polysomes (actively translated RNAs) and non-polysomes (free non-translated and poorly translated mRNAs) in a panel of colorectal cancer cells treated by 5-FU. This approach showed that 5-FU induces a translational reprogramming, characterized by an increased translational efficiency of specific genes that was mediated, at least in part, through 5-FU-modulation of miRNA expression.

## RESULTS

### Cells remain viable and metabolically active in response to 10 μM of 5-FU

**Figure 1:**
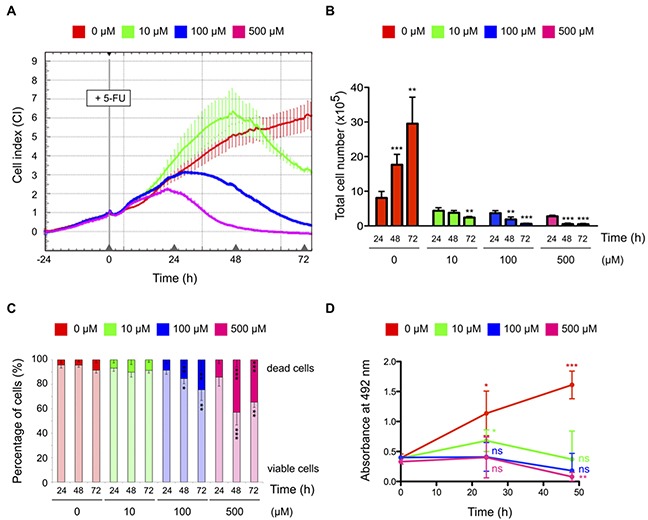
Effects of 5-FU treatment on HCT-116 cell biology **(A)** Real-time monitoring of HCT-116 cells in response to 5-FU treatment. Cells were treated 24 hrs post-seeding with increasing concentrations of 5-FU. The cell index (CI) relating change in cell number and attachment was monitored every 15 min for 72 hrs using the xCELLigence System and normalized to the starting time of 5-FU treatment. Delay in cytotoxicity is induced by 10 μM of 5-FU compared to 100 μM or 500 μM. This graph represents mean values of a quadruplicate, from one representative experiment. Experiments were repeated three times. Error bars indicate the standard deviation (SD). **(B-C)** Viability of HCT-116 cells in response to 5-FU treatment. Total cell numbers (B) and percentage of dead and viable cells (C) in response to 5-FU were quantified using trypan blue staining method at different time points. Compared to non-treated cells, exposition to 100 μM and 500 μM of 5-FU promotes drastic decrease in total cell numbers and increase in cell death, while exposition to 10 μM of 5-FU showed no impact on cell death. **(D)** Metabolic activity of HCT-116 cells in response to 5-FU treatment. Cells were treated with increasing concentrations of 5-FU and metabolic activity was analyzed using MTS assays at different time points. Compared to non-treated cells, metabolic activity was lost in cells treated with 100 μM or 500 μM of 5-FU, while a basal metabolic activity remains in cells treated with 10 μM of 5-FU. Graphs present means and SD of at least three independent experiments. *: *P* < 0.05; **: *P* < 0.01; ***: *P* < 0.001.

We first optimized 5-FU treatment condition to harvest HCT-116 colorectal cancer cells that retain cell viability and metabolic activity. Cell response to increasing concentrations of 5-FU was monitored in real-time for 72 hrs using electrical impedance-derived technology (Figure 1A) [25]. Compared to non-treated cells, two different kinetics of 5-FU response were observed depending on the dose. At 100 μM and 500 μM, 5-FU induced a decrease of impedance signal from 24 hrs post-treatment. At these doses, a significant reduction of total cell number and an increase in the percentage of dead cells were also observed (Figures 1B-1C). In response to 10 μM of 5-FU, the decrease of impedance signal was delayed by 24 hrs (Figure 1A). In this condition, the total cell number remained stable between 24 and 48 hrs post-treatment and no significant change in the percentage of dead cell was observed (Figures 1B-1C), while the 5-FU treatment was effective as soon as after 4 hrs of exposure as shown by the increased expression of the stress sensor p53 (Supplementary Figure 1A). These data showed that, compared to 100 μM and 500 μM, cytotoxicity in response to 10 μM of 5-FU was delayed, as cells were still viable at 24 and 48 hrs post-treatment.

To determine whether HCT-116 cells treated with different doses of 5-FU retained a metabolic activity at 24 hrs and 48 hrs, we performed MTS assays (Figure 1D). Cells treated with 100 μM and 500 μM of 5-FU showed a strong decrease of their metabolic activity. In contrast, a slight but significant increase in metabolic activity 24 hrs post-treatment was observed in 10 μM 5-FU treated cells, followed by a decrease back to their initial level by 48 hrs of treatment. Altogether, these data indicated that cells exposed to 10 μM of 5-FU remained viable and retained metabolic activity even at 48 hrs post-treatment.

### 5-FU reduces global protein synthesis

**Figure 2:**
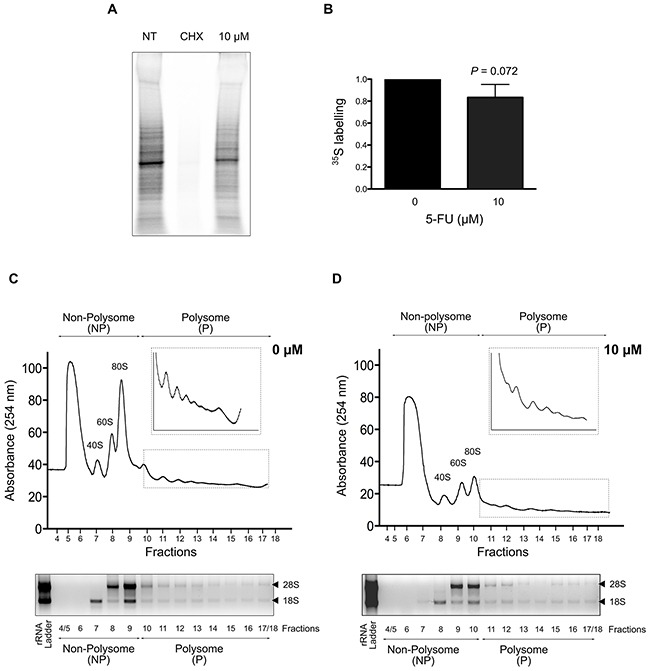
Impact of 5-FU treatment on protein synthesis in HCT-116 cells **(A-B)** Global protein synthesis in response to 5-FU. Protein synthesis was quantified by ^35^S labeling pulse-chase assays in non-treated and 5-FU treated cells. A representative gel is shown in (A) and mean quantification of three independent experiments is shown in (B). Compared to non-treated cells, a reproducible decrease in protein synthesis was observed in response to 10 μM of 5-FU for 24 hrs. Cycloheximide (CHX) was used as a positive control. **(C-D)** Polysome profiles in response to 5-FU. 40S and 60S ribosomal subunits, 80S monosomes and polysomes were separated by ultracentrifugation on sucrose gradients. One representative polysome profile of non-treated (C) and 10 μM 5-FU treated cells (D) is shown, as well as gel analysis of 18S and 28S rRNA used to verified RNA quality. On top of each profile, the fractions collected for microarray analyses (non-polysome NP and polysome P) are indicated. After RNA extraction, RNA quality was checked using bioanalyzer, the RNA Integrity Number (RIN) ranging from 6.6 to 9.3.

Before analyzing specific changes in translation that occur in response to 5-FU in viable cells retaining metabolic activity, we determined whether HCT-116 cells treated with 10 μM of 5-FU maintained their capacity to synthesize proteins. Using ^35^S pulse-labeling experiments, we compared levels of global protein synthesis between non-treated cells (NT) and cells exposed to 10 μM of 5-FU for 24 hrs (Figure 2A). Cycloheximide (CHX) treatment was used as a positive control of complete inhibition of global protein synthesis. ^35^S quantification revealed a decrease of about 20% of the global level of protein synthesis in treated cells compared to non-treated cells (Figures 2A-2B).

We then compared polysome profiles through sucrose gradients from non-treated cells and cells treated with 10 μM of 5-FU (Figures 2C-2D). Typical polysome profiles were obtained for non-treated and treated HCT-116 cells using both real-time absorbance detection and RNA visualization on agarose gel. The total quantity of 40S, 60S subunits, 80S monosomes and polysomes for a given amount of cytosolic extract was decreased in 5-FU treated cells. In particular, the 60S subunits and the monosomes 80S were drastically decreased. Using ^32^P pulse labeling, we confirmed that levels of ribosome decreased in 5-FU-treated cells compared to non-treated ones, and that 60S and 80S are more prone to reduction than 40S (data not shown). This decrease in ribosomes quantity is concordant with the reduction of ribosome production previously described in response to 5-FU [15, 18, 21]. Reduction in ribosome production in response to 5-FU was probably partly responsible for the decrease of global protein synthesis (Figures 2A-2B). These data showed that HCT-116 cells exposed to 10 μM of 5-FU for 24 hrs retained the capability to synthesize proteins although the global protein synthesis rate was reduced.

### 5-FU promotes association of a subset of mRNAs with polysomes

Because global protein synthesis was slightly decreased but still highly effective in response to 10 μM of 5-FU for 24 hrs in HCT-116 cells, we investigated whether this treatment induced a modulation of the translational efficiency of some specific mRNAs using translatome profiling, a widely used method [26]. We used a three-steps process that allows determining the variation of distribution of each cytosolic mRNA in non-polysomal fractions (NP, including free non-translated and poorly translated RNAs) and polysomal fractions (P, including actively translated RNAs) (Figures 2C-2D and Supplementary Figure 2A). First, for every gene, the detection of mRNA in non-polysomal (NP) and polysomal (P) fractions was assessed in non-treated and 5-FU treated cells using DNA microarray. Second, in each condition, the relative distribution of a given mRNA between NP and P fractions was estimated by calculating the ratio of the probe signal obtained for the P fraction to the NP fraction. Third, comparison of mRNA distribution between non-treated and treated cells was performed by calculating the translational index (TI), which represents the variation of the distribution of a particular mRNA between NP and P in response to 5-FU treatment. Thus, for each mRNA, the TI reflects the fold-change of its translation efficiency in response to 5-FU.

**Figure 3:**
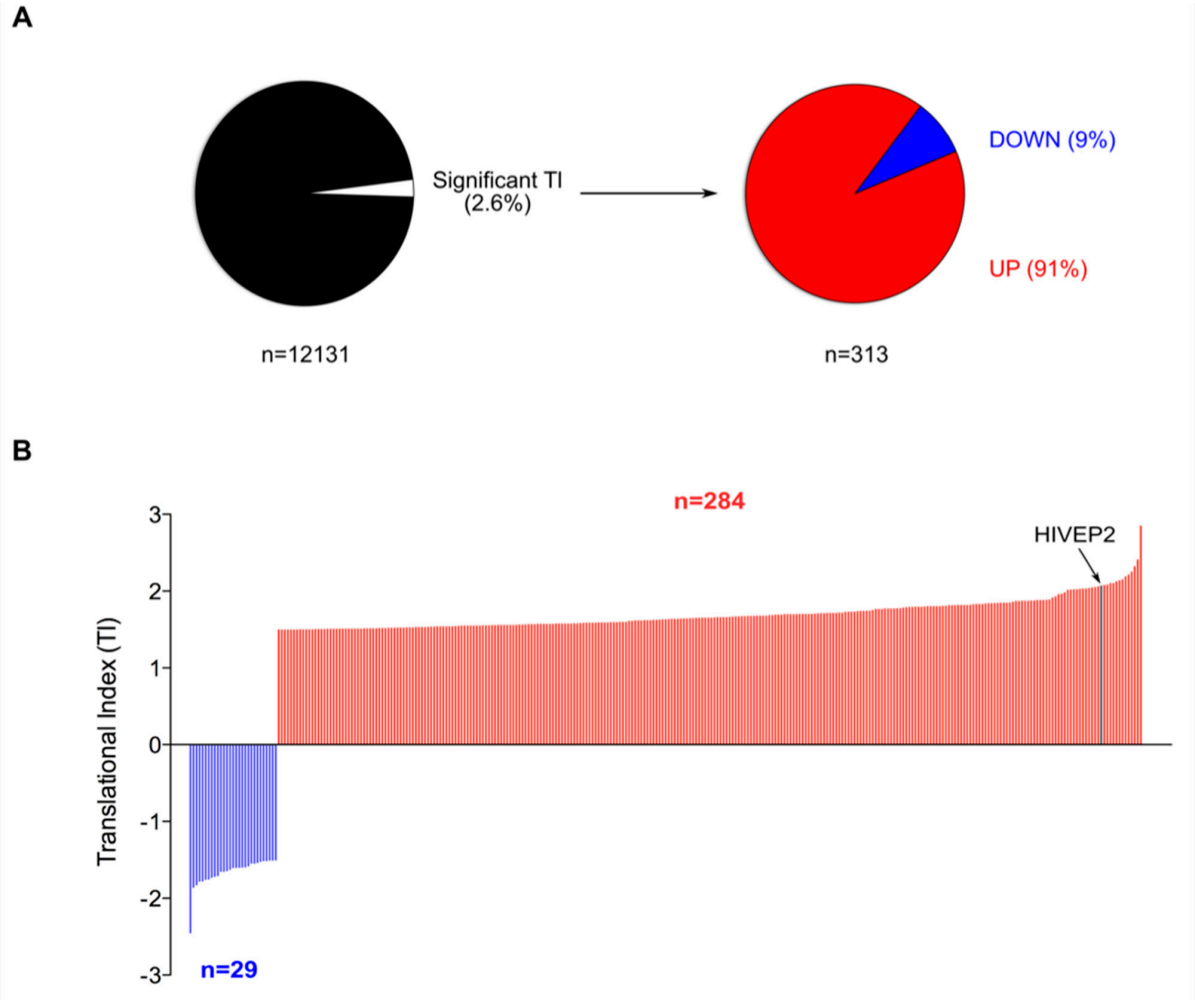
Effect of 5-FU on translatome of HCT-116 cells **(A)** Percentage of translationally deregulated mRNAs in response to 5-FU. Among the 12,131 genes commonly detected on the eight Affymetrix exon-arrays (NP-CTL, P-CTL, NP-5-FU, P-5-FU – each in duplicates), 2.6% (n=313) were significantly deregulated at translational levels (TI cut-off = 1.5, *P* < 0.05). Among these translationally deregulated genes, 9% were down-regulated and 91% up-regulated. These data were issued from two independent experiments. **(B)** Distribution of the Translational Index (TI). Among the 313 genes significantly deregulated at translational levels, TI varies from -2.5 to 2.9. TI of *HIVEP2* mRNA is indicated. For list of genes translationally deregulated see Table 1 and Supplementary Tables 1 and 2.

Two independent experiments were performed using this approach to determine change in TI in 10 μM 5-FU treated cells compared to non-treated cells. The probe intensities issued from the two biological replicates were significantly correlated supporting the robustness of this translatome profiling (Supplementary Figures 2B-2E). Globally, 12,131 genes were commonly detected in both non-treated and treated conditions (Supplementary Figure 2F). These common genes were used to analyze changes in translation efficiency in response to 5-FU by calculating the translational index (TI). By applying cut-off values of 1.5 and *P*-value < 0.05 to the TI of the 12,131 common genes [27], we identified 313 genes (2.6%) whose TI was significantly changed in response to 10 μM of 5-FU for 24 hrs in HCT-116 cells (Figure 3A and Supplementary Table 1). These data indicated that 5-FU treatment altered translation efficiency of some mRNAs. Among them, 29 were translationally down-regulated (9%) while 284 were up-regulated (91%). The range of TI varied from -1.5 to -2.5 and from 1.5 to 2.9 (Figure 3B and Supplementary Table 2). In particular, 25 genes displayed a TI above 2, indicating that 5-FU treatment induces a 2-fold increase in polysome association of some mRNAs (Table 1). Using lower or higher cut-off values (TI: 1.3 or 2.0), we observed that up-regulation of translation efficiency was always much more frequent than down-regulation (Supplementary Table 1). Thus, gene-specific modulation of translational efficiency in response to 10 μM of 5-FU for 24 hrs in HCT-116 cells corresponded mainly to stimulation (for genes list see Table 1 and Supplementary Table 2).

**Table 1:** List of the 25 most translationally dysregulated genes in response to 10 μM 5-FU in HCT-116 cells

Gene symbol	Translational Index	P-value
*Translationally up regulated mRNAs*		
SLC10A5	2.852	8.80E-07
SERTAD2	2.413	4.60E-09
FRK	2.325	0.00E+00
AC005035.1	2.254	0.00E+00
HIST1H2AM	2.216	4.19E-04
ANP32B	2.191	1.00E-10
C4orf14	2.153	0.00E+00
KLF7	2.142	4.16E-08
FAM123B	2.128	8.25E-08
MT-ND1	2.106	1.96E-03
C12orf5	2.105	0.00E+00
ZNF502	2.086	2.50E-04
RIPK2	2.078	0.00E+00
HIVEP2	2.073	0,00E+00
GPX2	2.064	9.81E-03
ZNF385B	2.052	0.00E+00
TRIM4	2.047	0.00E+00
MAP3K13	2.042	0.00E+00
C14orf126	2.035	6.00E-09
PLEKHM3	2.032	0.00E+00
MRPS18C	2.032	6.74E-04
CPEB4	2.024	0.00E+00
B3GALT1	2.024	8.81E-03
FAM200A	2.022	3.17E-06
GNPNAT1	2.018	0.00E+00
*Translationally down regulated mRNAs*		
CDKL4	−2.455	5.84E-05

**Figure 4:**
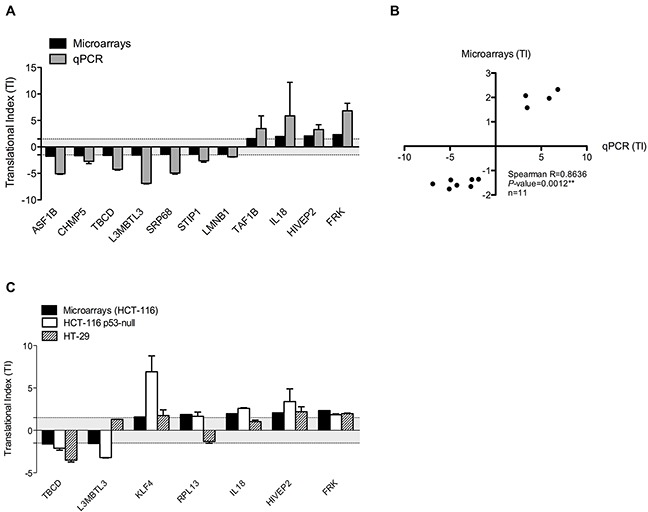
Validation of translatome profiling in a panel of colorectal cancer cell lines **(A)** Comparison of TI determined by Affymetrix exon-array and RT-qPCR in HCT-116 cell line. Validation in HCT-116 cells was performed by calculating the TI of 11 genes in response to 24 hrs exposure to 10 μM 5-FU using RT-qPCR on polysome profiles used for DNA microarrays analyses (two replicates) and additional, independent fraction preparation (at least two additional replicates). Similar down- and up-regulation were observed. **(B)** Correlation between TI calculated from Affymetrix exon-array and RT-qPCR in HCT-116 cells. Mean TI obtained by microarrays and RT-qPCR were plotted and correlation was assessed by Spearman correlation. A significant correlation was observed between Affymetrix exon-array and RT-qPCR data. **(C)** Comparison of TI in a panel of colorectal cancer cell lines in response to 10 μM of 5-FU for 24 hrs. TI of 7 genes determined by Affymetrix exon-array in HCT-116 cells (black bars) and determined by RT-qPCR in HCT-116 p53-null cells (white bars) and in HT-29 cells (striped bars) were compared. All the 7 genes showed similar translational modulation in both HCT-116 and HCT-116 p53-null cells. Only 3 genes out of 7 showed no translational dysregulation in HT-29 cells, whereas the 4 other genes showed similar translationnally up- and down-regulation in response to 24 hrs of 5-FU exposure in the three cell lines. Graph presents mean and SD. TI below cut-off used for microarrays analyses were represented by grey area.

Validation was performed by calculating the TI of 11 genes, which were selected on the basis of the significance and the absolute values of their TI measured using microarray. RT-qPCR were performed on HCT-116 NP and P fractions that were used for microarrays analyses (two independent replicates) and from two additional independent experiments. Compared to microarray data, similar up- and down-regulation of TI were observed in the panel of 11 genes analyzed by RT-qPCR (Figure 4A). A difference in the range of TI was observed between the two methods that can be explained by a difference in sensibility of these methods. However, a significant and positive correlation was observed between TI obtained from RT-qPCR and microarray analyses (Figure 4B). Similar analyses using RT-qPCR quantification of mRNA distribution within polysome profiles were performed in two additional colorectal cancer cells (Figure 4C). We used the p53-null HCT-116 cells and the mutant p53.R273H HT-29 cells (Supplementary Figure 1, IARC TP53 Database), to determine whether this translational regulation occurs in different colorectal cancer cell lines and whether it is dependent of p53 since several data support a role of p53 as a regulator of translational reprogramming [28]. Like in HCT-116 cells, TI of a panel of 7 genes were either reduced or increased in response to 5-FU in these two cell lines, similarly to what observed in HCT-116 cells. Of note only 3 genes in HT-29 did not show variation in their TI. Interestingly, since similar pattern of TI was observed in wild-type p53 HCT-116 cells, p53-null HCT-116 cells and mutant p53.R273H HT-29 cells, it appeared that changes in TI of each mRNA revealed by the translatome profiling are independent of p53. Altogether, the validation procedure performed on different genes in several cell lines confirmed that 5-FU treatment at 10 μM for 24 hrs reduced or increased the association of a subset of mRNAs with polysomes, indicating respectively either a reduction or an increase in translational efficiency of some mRNAs.

**Figure 5:**
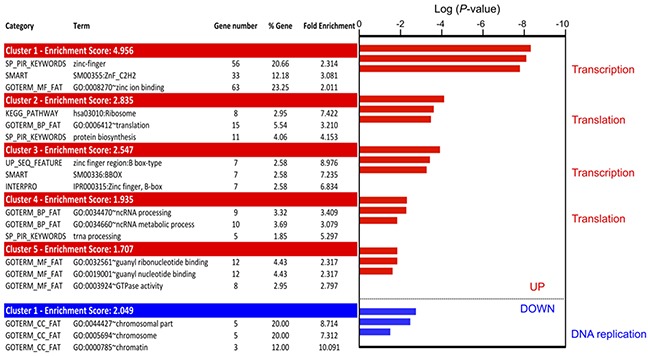
Gene ontology analysis of translationally deregulated genes in 5-FU treated HCT-116 cells GO enrichment was determined using the Functional annotation clustering tools from DAVID. Translationally up-regulated genes are mainly involved in transcription and translation, while translationally down-regulated genes are involved in DNA replication. Only the 3 first terms were shown for all clusters identified (up-regulation: 5 clusters; down-regulation: 1 cluster). For complete data see Supplementary Table 3.

To decipher whether alteration in translational efficiency directly resulted from 5-FU treatment or from 5-FU-induced change in transcriptional and post-transcriptional regulation, we quantified the levels of total and cytosolic mRNAs in response to 24 hrs of 10 μM 5-FU treatment in HCT-116 p53-null and HT-29 cells (Supplementary Figure 3). No concordance was observed between 5-FU-induced transcriptional, post-transcriptional and translational variation, as confirmed by correlation analyses. These data suggest that 5-FU alters translational efficiency independently of any change at mRNA levels. Finally, no change in TI analyzed in cytosolic lystates was observed in HCT-116 cells treated for 4 hrs by 10 μM 5-FU while increased in cytosolic mRNA levels were observed (Supplementary Figure 4). Once again, no concordance between transcriptional and translational events was observed at early time point although 5-FU affects more rapidly transcriptional regulatory events than the translational ones.

Altogether, our data showed that 5-FU treatment at 10 μM for 24 hrs alters the translatome independently of any transcriptional and post-transcriptional regulation of the targeted mRNA. In addition, 5-FU mainly increases the translation efficiency of a subset of mRNAs.

### Genes involved in DNA replication and gene expression regulation are selectively regulated by 5-FU at the translation level

To determine the main functions of genes whose recruitment whithin polysomes was altered in response to 5-FU in HCT-116 cells, we performed Gene Ontology analysis using the Functional annotation clustering analytic modules of DAVID bioinformatics resources that provides a rank classification of enriched functions based on determination of *P*-values and enrichment scores [29]. Statistical enrichment analyses were performed separately on genes translationally up- and down-regulated, using the lists of 313 (TI cut-off of 1.5, *P* < 0.05) and 798 (TI cut-off of 1.3, *P* < 0.05) translationally dysregulated genes. Clusters of functional annotation common to both lists are presented in Figure 5 (for a complete list of functional annotation clustering, see Supplementary Table 3).

**Figure 6:**
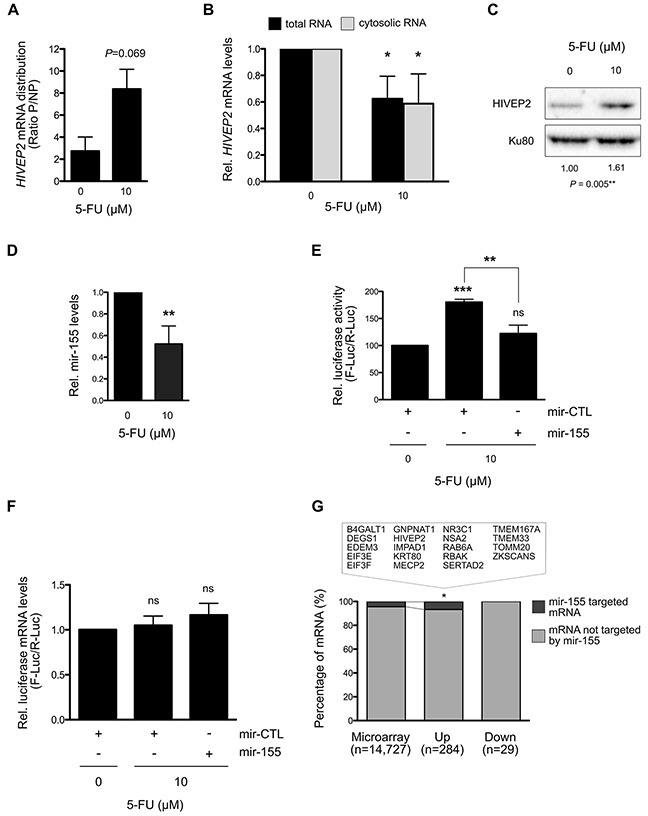
Translation regulation of *HIVEP2* mRNA by mir-155 through its 3′UTR under 5-FU treatment in HCT-116 cells **(A)** Distribution of *HIVEP2* mRNA in polysomal and non-polysomal fractions. *HIVEP2* mRNA levels was quantified in polysomal (mRNA actively translated) and non-polysomal fractions (free and poorly translated mRNA) by RT-qPCR in two additional and independent polysome separation. Increase in *HIVEP2* mRNA levels was observed in polysome in 5-FU treated HCT-116 cells compared to non-treated HCT-116 cells. **(B)** Variation of *HIVEP2* mRNA levels in response to 5-FU in HCT-116 cells. *HIVEP2* mRNA levels were quantified by RT-qPCR in both total and cytosolic RNA. 5-FU treatment significantly reduces *HIVEP2* mRNA levels. **(C)** Expression of HIVEP2 protein in response to 5-FU in HCT-116 cells. HIVEP2 protein levels were analyzed by Western blot. 5-FU treatment increases HIVEP2 protein levels. **(D)** Expression of mature mir-155 under 5-FU treatment. Mature mir-155 expression levels were quantified by RT-qPCR in treated and untreated HCT-116 cells. 5-FU treatment significantly reduces mature mir-155 expression levels. **(E-F)** Analysis of HIVEP2 translational regulation through its 3′UTR using luciferase reporter assays. Luciferase activities were measured to determine the role of mir-155 on HIVEP2 3′UTR-dependent translation in response to 0 or 10 μM 5-FU for 24 hrs (E). mRNA levels of *Firefly* and *Renilla* genes were analysed to verify that variation in luciferase activity shown as F-Luc/R-Luc (RLU) is not due to variation of F-Luc/R-Luc at mRNA levels (F). **(G)** Enrichment of mir-155 target genes in translationally dysregulated genes. Statistical analyses showed that mir-155 target genes were enriched in translationally up-regulated genes in response to 5-FU. The list of mir-155-target genes translationaly up-regulated is given in the box. Graphs present means and SD of at least two independent experiments. *: *P* < 0.05; **: *P* < 0.01; ***: *P* < 0.001.

Using this approach, only one functional cluster was identified for the genes whose translation efficiency was down-regulated in response to 5-FU treatment. This cluster contained 5 of the 29 translationally down-regulated genes (17%). It was composed of genes involved in DNA replication, such as ASF1B or TBCD (Figure 5 and Supplementary Table 3). These data suggested that 5-FU reduced the translation efficiency of mRNAs involved in DNA replication.

Genes whose translation efficiency was up-regulated by 5-FU clustered into five groups showing enrichment scores ranging from 1.7 to 4.9 (Figure 5). The group which displayed the most significant *P*-value and the highest enrichment score, contained genes encoding zinc finger proteins, including several ZNF family members and HIVEP2 (Figure 5 and Supplementary Table 3). These proteins are mainly involved in transcription regulation. A second cluster closely related to the transcription process (cluster 3) was enriched in genes coding proteins with B-box domains. These data suggest that 5-FU treatment selectively increases the translation efficiency of a subset of mRNAs encoding transcriptional regulators. Translationally up-regulated genes were also enriched in genes involved in translation and translational regulation. Indeed, clusters 2 and 4 contained genes involved in translation initiation and elongation (*EIF3F*, *EIF3E*…) and in production of the translational machinery, including ribosome components (*RPL13*…) and tRNA maturation factors. Altogether, these data showed that genes whose translation efficiency was up-regulated in response to 5-FU treatment for 24 hrs were mainly involved in regulation of gene expression, including transcription and translation.

### 5-FU induces translational up-regulation of *HIVEP2* mRNA through down-regulation of mir-155

To investigate the molecular mechanisms contributing to the modulation of translational efficiency of specific mRNA in response to 5-FU treatment, we focused our attention on *HIVEP2* mRNA, one of the most translationally deregulated genes (Figure 3B). Indeed, our translatome profiling and validation process showed that the distribution of *HIVEP2* mRNA was significantly increased in polysomes compared to non-polysomes in response to 5-FU in a panel of three colorectal cancer cell lines (Figures 3B, 4A, 4C and 6A). We determined whether this change in *HIVEP2* mRNA translation was paralleled with a change in mRNA and protein levels in HCT-116 cells. RT-qPCR analysis showed a significant 40% decrease of *HIVEP2* mRNA levels in both total and cytosolic extracts in response to 5-FU treatment (Figure 6B). In parallel, a significant 1.5-fold increase in HIVEP2 protein levels was observed in response to 5-FU treatment (Figure 6C and Supplementary Figure 5A). These data demonstrated that despite the decrease of *HIVEP2* mRNA levels in response to 5-FU, the selective increase in its translational efficiency led to an increase in HIVEP2 protein levels.

miRNAs reduce mRNA translation by direct interaction with specific mRNAs that is a pre-requisite for subsequent mRNA degradation [30, 31]. *HIVEP2* mRNA was previously identified as a direct target of mir-155 [32]. We thus wondered whether mir-155 expression may be regulated by 5-FU, thereby participating in the stimulation of *HIVEP2* mRNA translational efficiency. In HCT-116 cells, quantification of mir-155 expression by RT-qPCR showed a significant reduction of 50% in response to 5-FU treatment for 24 hrs (Figure 6D). Thus, by decreasing mir-155 expression, 5-FU could counteract mir-155-mediated inhibition of *HIVEP2* translation and thus promote *HIVEP2* mRNA translation. Interestingly, reduction in mir-155 levels was observed from 4 hrs (Supplementary Figure 5B), suggesting a direct effect of 5-FU on mir-155 expression. To determine the effect of mir-155 on *HIVEP2* mRNA translation, we performed 3′UTR luciferase reporter assays using HIVEP2 3′UTR reporter. Over-expression of mir-155 in the absence of 5-FU significantly reduced the Firefly/Renilla luciferase activity ratio of the HIVEP2 3′UTR reporter and of the positive control BACH1 3′UTR reporter while it had no impact on a 3′UTR-less negative control (Supplementary Figure 5C) [32]. Using this system, we assessed the role of HIVEP2 3′UTR and mir-155 expression in the increase in *HIVEP2* mRNA translation in response to 5-FU treatment (Figure 6E). In the absence of transient mir-155 over-expression (mir-CTL condition), 5-FU treatment significantly increased the Firefly/Renilla luciferase activity ratio of the HIVEP2 3′UTR reporter. Importantly, this increase in luciferase activity was not paralleled with a variation in luciferase mRNA levels (Figure 6F). These data suggested that increase in luciferase activity results from change in translation rather than change in transcription of the chimeric Firefly Luciferase – HIVEP2 3′UTR. Thus, *HIVEP2* mRNA translation in response to 5-FU treatment was, at least in part, related to the 3′UTR of *HIVEP2* mRNA. In 5-FU treated cells, mir-155 expression was then restored using an expression vector (Figure 6E). Restoration of mir-155 expression reduces the induction of Firefly/Renilla luciferase activity ratio in 5-FU treated cells. In addition, no significant change in the luciferase mRNA levels was observed, indicating that restoration of mir-155 in 5-FU treated cells inhibits translation without affecting transcriptional expression of the luciferase reporter assay (Figure 6F). Interestingly, similar reduction in mir-155 expression from 4 hrs post-treatment and in mir-155-dependent HIVEP2 translation in response to 5-FU was observed in p53-null HCT-116 cells, indicating a p53-independent effect (Supplementary Figure 6). Thus, over-expression of mir-155 was sufficient to inhibit the increase in *HIVEP2* mRNA translation that was induced by 5-FU. Altogether, these data showed that the alteration of mir-155 in response to 5-FU mediated a translational regulation of the *HIVEP2* mRNA.

To determine whether mir-155 may have a broader role in translational regulation in response to 5-FU treatment, we first analyzed the enrichment of mir-155 target genes among the genes whose translation efficiency was altered by 5-FU treatment in HCT-116 cells, using a list of 719 experimentally validated mir-155 target genes (miRTarBase [33]) (Figure 6G and Supplementary Table 4). Interestingly, a significant enrichment in mir-155 target genes was observed among the translationally up-regulated genes (Up) when compared to the whole genome (Up vs Microarray, *P* = 0.0389), while no enrichment of mir-155 target genes was found among the genes translationally down-regulated by 5-FU (Down vs Microarray, *P* = 0.3088). As shown in Figure 6G, 19 of the genes that were translationally up-regulated by 5-FU corresponded to mir-155 target genes (6.7%). We validated the translationnally up-regulation of a panel of mir-155 target genes in response to 5-FU in HCT-116 p53-null and HT-29 cells. Although the kinetic of mir-155 expression in response to 5-FU exhibited opposite variation at 24 hrs in HT-29 compared to the isogenic HCT-116 cellular model while this kinetic remained similar at 4 hrs (Supplementary Figures 5B, 6A and 7), the three cell lines showed a comparable tendency to translationally up-regulation of mir-155 target genes in response to 5-FU (Supplementary Figure 8). These data suggest that the early down-regulation of mir-155 in response to 5-FU treatment may contribute to the increased translation of a subset of mRNAs in colorectal cancer cells. Interestingly, Gene Ontology analysis of these mir-155 target genes whose translation was up-regulated in response to 5-FU mainly corresponded to proteins involved in transcription (Supplementary Table 5).

## DISCUSSION

The anti-metabolite 5-FU has been shown to alter gene expression at transcriptional and splicing levels and to alter the translation machinery, mainly by affecting the processing and functions of rRNA and tRNA [15, 18, 19, 21, 22]. However, the impact of 5-FU on translational control remained unclear. Using translatome profiling, we show here for the first time that 5-FU induces a translational reprogramming since, while reducing the global protein synthesis, 5-FU increases translation of a subset of mRNAs, at least in part through miRNA-based mechanisms.

As already reported, we identify two kinetics of cellular response to 5-FU [7]. While high doses of 5-FU exposure result in drastic and rapid cytotoxic effects, low doses, which correspond to doses used in the clinic [34, 35], promote delay in cytotoxicity. In particular, cells exposed to 10 μM of 5-FU for 24 hrs remain viable and metabolically active, and keep their capacity to synthesize proteins. By comparing mRNA levels in polysomal (translated mRNAs) and non-polysomal (free and poorly translated mRNAs) fractions using DNA microarray [26], we demonstrate that low doses of 5-FU specifically alter the translation efficiency of about 300 mRNAs. Importantly, the validation procedure performed on different genes in a panel of three colorectal cancer cell lines confirmed that 5-FU treatment alters the association of some mRNAs with polysomes. By analyzing the abundance of mRNAs in polysomal fractions using genome-wide approaches two previous studies have suggested that 5-FU affects translation [23, 24]. However, since no normalization to non-polysomal fraction or total RNA was done in these studies, alteration of translation efficiency was not directly addressed in these studies. In addition, both studies had potential bias. Indeed, one of these studies used colorectal cancer cells with decreased or increased expression of thymidylate synthase, a key mediator of 5-FU cytotoxicity, thus complicating the analysis of the direct impact of 5-FU on translation [7, 23]. The second study used Hsp70 immunoprecipitation to purify ribosomes presenting newly synthesized proteins [24]. This approach may suffer from several biases since Hsp70 was recently reported to bind RNA, Hsp70 is not required for newly synthesized small proteins, and Hsp70 levels are reduced in response to 5-FU [36–39].

Our data indicate that exposure to 5-FU promotes increase in translational efficiency of only a subset of mRNAs. It appeared that the 5-FU-induced change in translation efficiency obtained in our experimental conditions is independent of any transcriptional and post-transcriptional modulation induced by 5-FU treatment in a panel of three colorectal cancer cell lines. Indeed, we showed that, although change in translation efficiency is not an early event, modulation of translation does not correlate with modulation of transcription, as shown for several genes including *HIVEP2*. Change in transcription can thus not explain the observed change in translation. Several mechanisms can explain the change in translational control of only a subset of transcripts in response to 5-FU. Such modulation in translational efficiency can result for example from 5-FU-induced alteration of expression of factors involved in translational control such as proteins or miRNAs [2]. Our data showed that 5-FU-induced change in translational efficiently occurred independently of the p53 protein, a stress sensor known to regulate translation through numerous mechanisms [28]. Our data rather identified mir-155 as a common regulator of 5-FU-mediated alteration of translational efficiency for several target genes.

We indeed showed that reduction of mir-155 in response to 5-FU can promote *HIVEP2* mRNA translation in HCT-116 colorectal cancer cells. In addition, determination of the molecular mechanism by which 5-FU regulates *HIVEP2* also supports that 5-FU regulates translation efficiency. Indeed, cell exposure to low doses of 5-FU decreases *HIVEP2* mRNA levels in both total and cytosolic fractions while in the same time, *HIVEP2* mRNA is concentrated in the actively translated polysomal fractions, and HIVEP2 protein levels are increased in 5-FU treated cells. Moreover, our data reveal that increased translation of *HIVEP2* mRNA in response to 5-FU results from abolition of mir-155-mediated inhibition of *HIVEP2* mRNA translation. By reducing mir-155 expression, 5-FU promotes translation of *HIVEP2* mRNA. This observation is in accordance with the repressing role of miRNAs, which have been shown to inhibit translational regulation before inducing mRNA degradation – inhibition, which can be removed by reducing miRNA expression [30, 31]. Furthermore, we showed and validated that mir-155 target genes are enriched in genes translationally up-regulated, establishing that reduction in mir-155 levels in response to 5-FU could explain about 7% of the translationally up-regulated gene. Interestingly, plasmatic mir-155 expression levels were found to be decreased after 5-FU-based chemotherapy exposure of colorectal cancer cells’ patients [40]. miRNA regulation is likely a more general mechanism leading to alteration of translation in response to 5-FU, because miRNA profiling identified several miRNAs, whose expression is either up- or down-regulated in response to 5-FU [12, 14, 41]. However, until now, these changes in miRNA expression levels have not been linked to alteration of translation. Additional mechanisms remain to be explored to explain the translational control of a specific subset of mRNAs in response to 5-FU treatment. In particular, the impact of 5-FU incorporation into RNAs of the translational machinery on translational control remains to be investigated.

We show that the selective translation of mRNAs in response to 5-FU is accompanied by a decrease in global protein synthesis. This reduction in protein synthesis could result from the previously described reduction in pre-rRNA processing in response to 5-FU since a strict correlation occurs between the rate of ribosome biogenesis and protein synthesis [18, 42, 43]. Therefore, the concomitant decrease in global protein synthesis and increase in translation of a selected subset of mRNAs indicate that 5-FU promotes translational reprogramming in colorectal cancer cells.

In conclusion, in the present study we provide a novel mechanism, which supports the importance of translational control in 5-FU-induced cellular response. Overall, our data indicate that low doses of 5-FU promote translational reprogramming of colorectal cancer cells. The gene-specific stimulation of translation induced by 5-FU involves at least in part miRNA-dependent translational regulation. However, additional mechanisms remain to be investigated in the future. Altogether, our data add to the growing body of evidences that support the direct contribution of translation in establishing anti-cancer drug response as well as potential treatment failures.

## MATERIALS AND METHODS

### Cell culture

Human colorectal cancer HCT-116 and HT-29 cells were obtained from ATCC (CCL-247, HTB-38). Cells were maintained in DMEM-glutaMAX supplemented with 10% FBS and 1% penicillin/streptomycin (Life technologies) at 37°C under 5% CO_2_ atmosphere. 5-Fluorouracil (5-FU) was kindly provided by Centre Léon Bérard (purchased at Sanofi-Aventis), stored at room temperature at 384 mM in sterile water and diluted in cell culture medium.

### Cell proliferation and viability

Real-time cell behavior was monitored using xCELLigence RTCA system (Roche), which allows label-free and dynamic monitoring of cells by measuring electrical impedance. 10^5^ cells were seeded in 96 E-plates (Roche) 24 hrs prior 5-FU treatments. RTCA system displays the measurements of impedance signal as Cell Index (CI) values, providing quantitative information about the different biological status of the cells including number, viability, proliferation and mobility. CI values curves were normalized to the time point of 5-FU administration. MTS were performed at different time points on 10^5^ cells seeded into 96 well-plate 24 hrs prior 5-FU treatments using Cell Titer Aqueous One Solution Cell Proliferation assays as described by the manufacturer (Promega). Cell viability and total cell numbers were quantified in response to 5-FU treatment by trypan blue staining method using Cedex XS analyzer (Roche) from 150.10^5^ cells seeded in 24 well-plates 24 hrs prior 5-FU treatment.

### Global protein synthesis

Cells were plated 72 hrs before labeling in normal medium and treated with 10 μM 5-FU for 24 hrs before labeling. Normal medium was replaced with methionine-cysteine free DMEM for 30 min before labeling. [^35^S]-methionine-cysteine was added in the medium at 75 μCi/mL and cells were incubated for 1 hr at 37°C. Labeling was stopped by washing cells with ice cold 1X PBS. Cells were scraped in SDS-PAGE laemmli sample buffer, and incubated 10 min at 95°C. Fifteen μg of protein was loaded onto a 4-20% gradient SDS-PAGE. [^35^S]-met-cys incorporation was quantified using PhosphorImaging on a FLA-9500 apparatus (GE). For each labeling experiment, protein quantification and SDS-PAGE were performed in duplicate.

### Preparation of mRNA-associated polysomes

Cells were seeded at 10^7^ cell/15 cm dish and treated for 24 hrs with 5-FU. Cells were then incubated for 15 min with 100 μg/ml cycloheximide (CHX) and washed twice with cold 1X PBS-100 μg/ml CHX before harvesting. Cytosolic lysates were prepared in lysis buffer (20 mM KCl, 5 mM MgCl_2_, 50 mM Tris-HCl pH 7.4, 4.5% sucrose, 0.5 mM DTE and 100 μg/ml CHX) using dounce homogenizer and two successive 10 min centrifugations at 4°C at 1,000 and 12,000 x *g*. Ten-40% sucrose gradients were prepared using four solutions (10%, 20%, 30% and 40% of sucrose dissolved in lysis buffer) that were introduced one by one in increased concentration order after 30 min at -80°C in Ultra-Clear Tube 9/16 × 3 ½ (Beckman). One mg of cytosolic proteins was loaded onto a sucrose gradient defrozen overnight at 4°C and sedimented by ultra-centrifugation at 40,000 rpm for 2 hrs at 4°C using SW41 rotor on L7-55 ultracentrifuge (Beckman). The gradients were collected in 18 fractions and absorbance profiles were generated at 254 nm using an ISCO UA-6 detector. RNA quality of the different fractions was checked on agarose gel. Non-polysomal or polysomal fractions were pooled for further RNA extraction using Trizol LS Reagent as described by the manufacturer (Life technologies).

### Affymetrix exon-array

Quality of RNA purified from polysome fractionation was verified using BioAnalyzer (Agilent) (RIN ranging from 6.6 to 9.3). 250 ng of total RNA was processed with the GeneChip WT Sense Target Labeling kit and hybridized to GeneChip Human Exon 1.0 ST arrays. Affymetrix exon-array data were normalized with quantile normalization. Antigenomic probes were used to perform the background correction. Only probes targeting exons annotated from full-length cDNA were retained for analysis. Cross hybridizing probes and probes with lower signals intensity than anti-genomic background probes showing the same GC content were removed. Only probes with a DABG *P*-value ≤ 0.05 in at least half of the arrays were considered for further statistical analysis. Arrays were performed in two independent replicates. The median intensity of all constitutive exonic probes was calculated for each gene in each sample, and the experimental samples and control groups were compared using a Student's paired t-test. The adopted strategy to identify the translationally regulated genes depends on calculating the translational index (TI) (Supplementary Figure 2A). First the ratios of active mRNAs (polysomal fraction P) to inactive mRNAs (non-polysomal fraction NP) was calculated for both 5-FU treated and non-treated cells. Then the translational index (5-FU P/NP)/(Control P/NP) presents the translational changes for each individual transcript. List of genes significantly deregulated at translational levels was determined using Student's paired t-test (*P* < 0.05) and TI cut-off (1.3, 1.5 or 2) that are classical values used in translatome analyses [27]. Gene ontology was performed on gene lists using the online DAVID tools using Functional annotation clustering analytic modules that performed dedicated statistical analysis [29]. The Affymetrix exon-array data reported in this article have been deposited in NCBI's Gene Expression Omnibus (GEO) database (accession number GSE77180).

### Real-time quantitative RT-qPCR

The Affymetrix exon-array was validated by RT-qPCR using RNA purified from the two polysomal profiles used for the exon-array analyses and from four additional and independent polysomal profiles. Three criteria have been used to select genes for validation: a significant difference in mRNA distribution within polysomal and non-polysomal fractions between non-treated and 5-FU-treated cells; a Translational Index (TI) corresponding to the highest TI variations; and a mix of translationnally up- and down-regulated genes. 250 ng of total RNA were reverse transcribed using the M-MLV RT kit and random primers (Invitrogen), according to the manufacturer's instructions. Quantitative real-time PCR (qPCR) was carried out using the Light cycler 480 II real-time PCR thermocycler (Roche). Expression of mRNAs was quantified using LightCycler 480 SYBR Green I Master Mix (Roche) (Supplementary Table 6) and normalized using *GAPDH* expression according to the 2^−ΔΔCt^ method. Reverse transcription of miRNAs was performed using TaqMan MicroRNA RT kit (no. 4366596, Life Technologies). miRNA RT-qPCR was carried out using 7900HT Fast Real-Time PCR System (Applied Biosystems). The expression of mature miR-155 and RNU6B was assessed using qPCR primer sets (Life Technologies, cat.no. 4427975-002623 and 4427975-001093, respectively).

### Western blot

Cells were washed in 1X PBS, harvested and total proteins were directly extracted in Laemmli buffer (62.5 mM Tris-HCl pH 6.8, 1% SDS, 0.1 M DTE). Twenty μg of total proteins were separated on a 12 % SDS-PAGE and transferred on a nitrocellulose membrane using a semi-dry transfer apparatus. Membranes were saturated with 5% milk and incubated with mouse monoclonoal antibodies against HIVEP2 (ab70599, AbCam), p53 (DO-1, ab1101, AbCam) and Ku80 (ab87860, AbCam). Detection was performed with the secondary anti-mouse antibody (A4416, Sigma) with Clarity Western ECL substrat kit using ChemiDoc Imager (BioRad).

### Luciferase reporter assays

500 ng of either miRNA control plasmid (pMSCV-puro-GFP-mir-CTL) or miR-155 expression vector (pMSCV-puro-GFP-mir-155) was co-transfected with 200 ng of either HIVEP2 reporter plasmid (pMIR-REPORT-dCMV 3′ UTR HIVEP2) or control reporter plasmid (pMIR-REPORT-dCMV) into HCT-116 or HCT-116 p53-null cells using Lipofectamine reagent (Invitrogen) following the manufacturer's procedure. Firefly luciferase activity was used to monitor 3′UTR activity while Renilla luciferase activity was used as a control of transfection efficiency. 10^5^ cells were seeded 24 hrs prior transfection and were treated with 5-FU 24 hrs post-transfection for additional 24 hrs before analyzing luciferase activity using Dual Reporter Luciferase Assays (Promega). The plasmids are a kind gift of Dr. Erik K. Flemington laboratory.

### Statistical analyses

Statistical analyses were performed using GraphPad Prism 5.0a (GraphPad Software, Inc). Mean comparison was performed using Student *t*-test. Correlation between translational index (TI) issued from DNA microarrays and RT-qPCR was tested using the non-parametric Spearman r test. Enrichment of mir-155 target genes was investigated by Chi-square test. All *P*-values corresponded to two-tailed *P*-values. *P*-values <0.05 were considered statistically significant. Statistical results are given on the graphs using conventional annotations: *: *P*<0.05; **: *P*<0.01; ***: *P*<0.001.

## SUPPLEMENTARY FIGURES AND TABLES







## Abbreviations

5-FU: 5-Fluorouracil
CHX: cycloheximide
miRNA: microRNA
mRNA: messenger RNA
rRNA: ribosomal RNA
tRNA: transfert RNA
