# Supplementary material for: Translational reprogramming of colorectal cancer cells induced by 5-fluorouracil through a miRNA-dependent mechanism
Source: Oncotarget. 2017 May 3;8(28):46219–33. doi: 10.18632/oncotarget.17597 (PMC5542262; doi:10.18632/oncotarget.17597)
Supplement: Supplementary file 1 [file oncotarget-08-46219-s001.pdf]

Translational reprogramming of colorectal cancer cells induced by 5-fluorouracil through a miRNA-dependent mechanism

SUPPLEMENTARY FIGURES AND TABLES

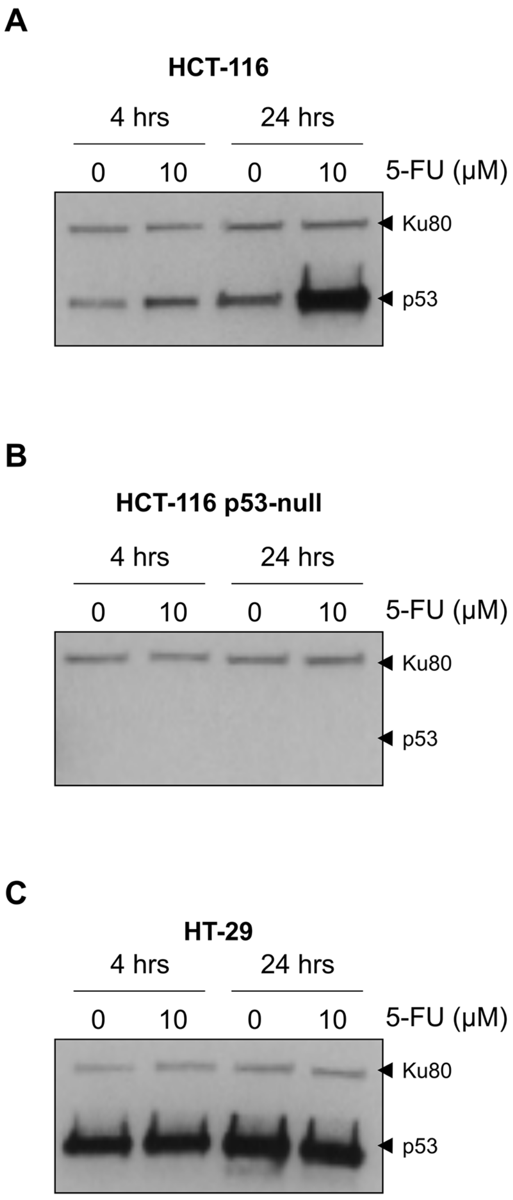

Supplementary Figure 1: Expression of p53 in response to 5-FU. Levels of p53 protein were analysed by Western blot in response to 10 µM of 5-FU exposure for 4 hrs and 24 hrs in three human colorectal cancer cell lines: HCT-116 cells expressing a wild-type p53 protein **(A)**; HCT-116 p53-null cells depleted from p53 protein **(B)**; and HT-29 cells expressing a mutant p53 protein **(C)**. A 2-fold increase in p53 protein levels was observed in HCT-116 cells treated with 10 µM of 5-FU for 4 hrs until reaching a 5-fold increase at 24 hrs post-treatment. These data indicate that 5-FU treatment was successful in HCT-116 cells even at early time point and low concentration of 5-FU. In contrast, no change in p53 protein levels was observed in p53-null and mutant p53 cells, as expected.

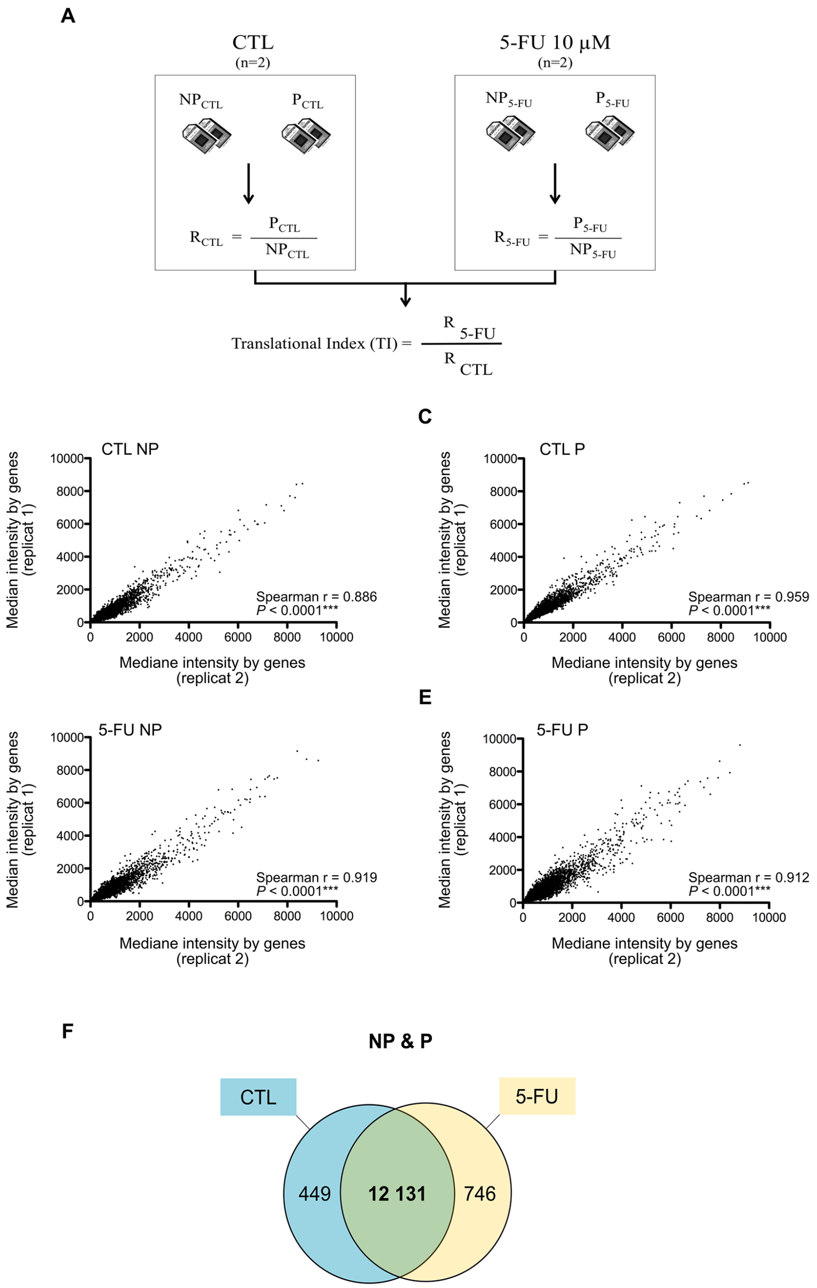

Supplementary Figure 2: Gene detections using Affymetrix exon-array. (A) Workflow of the genome-wide analysis of translatome in response to 5-FU treatment. Impact of 10 μM 5-FU on translational status of each mRNAs was determined using Affymetrix exon-arrays by calculating the translational index, which compared levels of mRNA-associated polysomes (polysomal fraction, P) to the ones of mRNA non-associated to polysomes (non-polysomal fraction, NP) in non-treated (CTL) and 5-FU treated cells. This analysis was performed in two independent experiments. **(B-E)** Correlation of the gene median intensities between the two biological replicates. Median intensities of all the mRNAs detected by Affymetrix exon-array were compared between biological replicates 1 and 2 for the non-treated non-polysomal fractions (CTL NP, B), the non-treated polysomal fractions (CTL P, C), the 5-FU treated non-polysomal fractions (5-FU NP, D) and the 5-FU treated polysomal fractions (5-FU P, E). Each correlation showed a strong Spearman r (> 0.89) and a significant p-value ($P < 0.0001$), supporting the robustness of the genome-wide analyses based on the two biological replicates. **(F)** Venn diagrams comparing gene detections in non-treated and 5-FU treated conditions. In both non-polysomal and polysomal fractions, 12,131 genes were commonly detected in non-treated (CTL) and 5-FU conditions. In contrast, 449 genes were detected in CTL conditions but not in 5-FU conditions, while 746 genes were detected only in the 5-FU conditions.

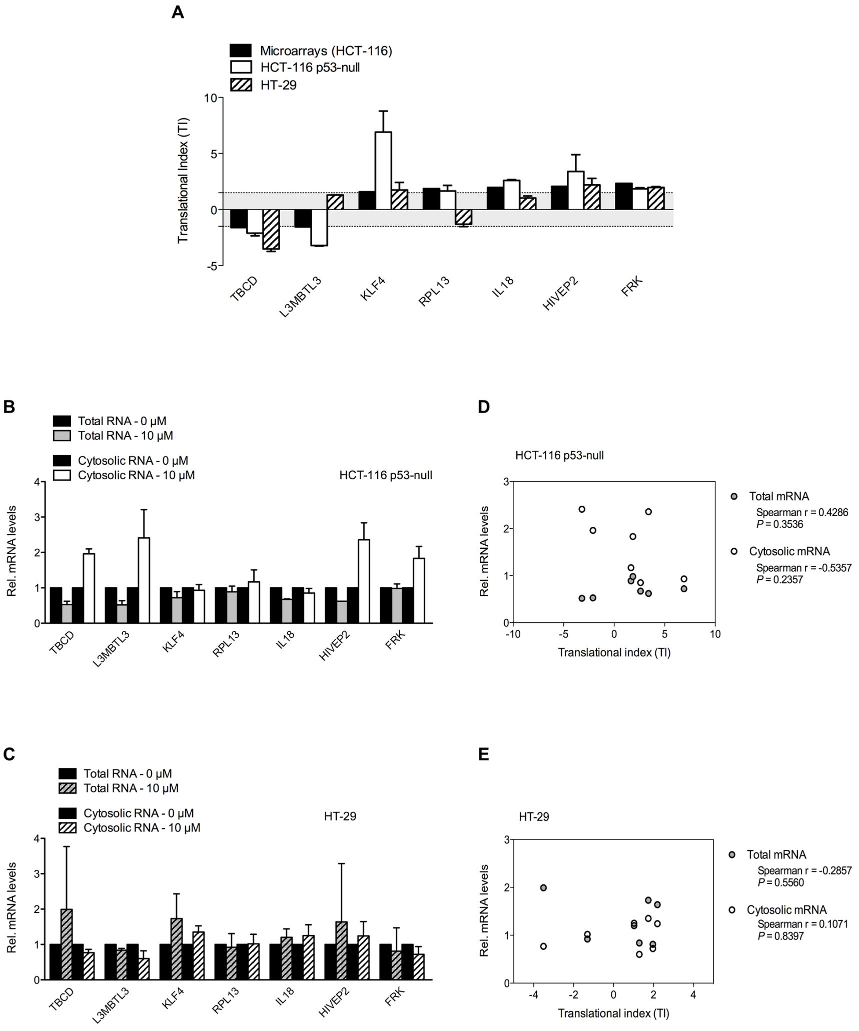

Supplementary Figure 3: Absence of correlation between translation and transcription in response to 24 hrs of 5-FU exposure in HCT-116 p53-null and HT-29 cells. **(A)** Comparison of Translational Index (TI) in three colorectal cancer cell lines. Translational Index (TI) of 7 genes that were determined in response to 24 hrs of 10 µM 5-FU treatment in HCT-116 cells using microarrays (black bars) were compared to the ones determined by RT-qPCR in HCT-116 p53-null cells (white bars) and HT-29 cells (striped bars). This graph is identical to the one shown in Figure 4C and shows that the translational modulation in response to 5-FU treatment was similar in the three cell lines. **(B-C)** Variation of mRNA levels in response to 5-FU. In response to 24 hrs of 10 µM 5-FU, levels of the 7 mRNAs of interest that were purified from either total or cytosolic lysates of HCT-116 p53-null cells (B) or HT-29 cells (C) were quantified using RT-qPCR. For most of the mRNAs, no concordant variation was observed in total and cytosolic lysates, indicating that 5-FU exposure affects not only transcriptional but also post-transcriptional regulation. **(D-E)** Comparison of Translation Index (TI) and relative mRNA levels. Using the 7 genes of interest, Translational Index (TI) and relative mRNA levels measured in total and cytosolic lysates of either HCT-116 p53-null cells (D) or HT-29 cells (E) in response to 5-FU exposure were compared. No significant correlation was observed between TI and mRNA levels, suggesting that variation of translation does not directly result from variation in transcriptional or post-transcriptional events, which affect the mRNA of interest. Graphs present means and SD of two independent experiments. *: $P < 0.05$; **: $P < 0.01$; ***: $P < 0.001$.

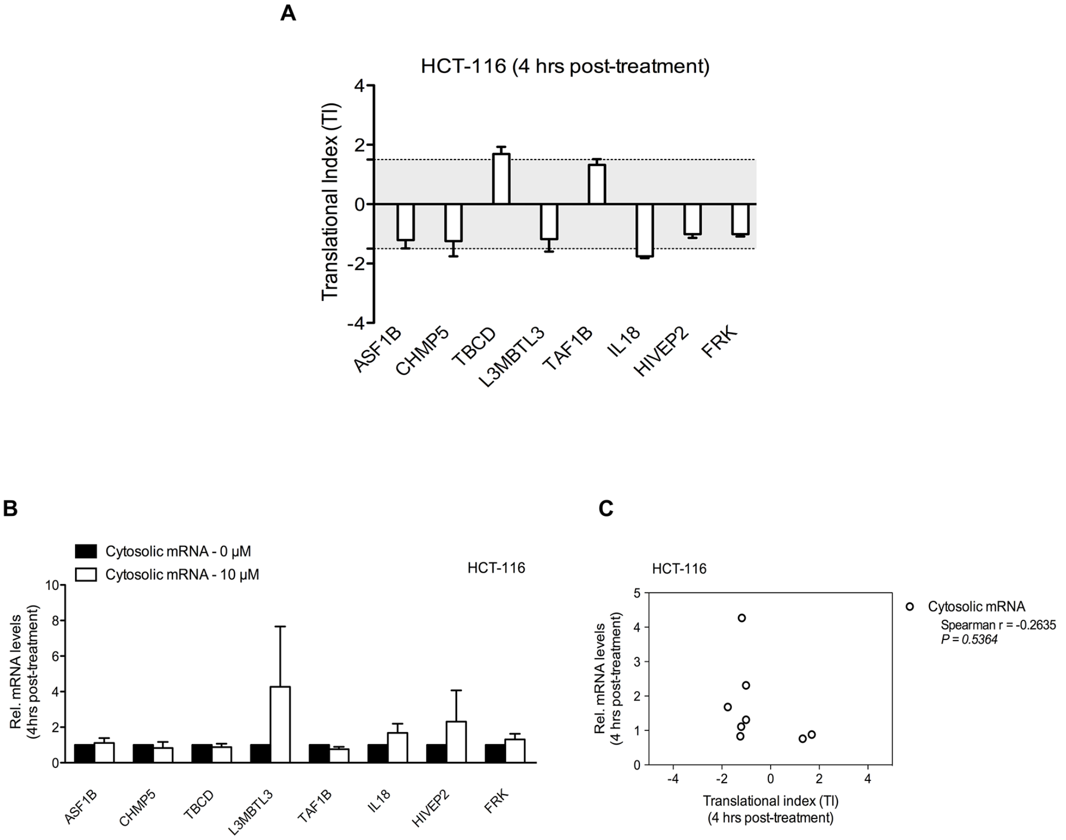

Supplementary Figure 4: Absence of correlation between translation and transcription in response to 4 hrs of 5-FU treatment in HCT-116 cells. (A) Translational Index (TI) in response to 4 hrs of 10 µM 5-FU. Translational Index (TI) of 8 genes was determined in response to 4 hrs of 10 µM 5-FU treatment in HCT-116 cells using RT-qPCR. Using similar threshold as for microarrays, none of the 8 genes showed variation in translation, TBCD and IL18 having only a borderline variation in their TI. These data suggest that translation is not altered in HCT-116 cells exposed to 10 µM of 5-FU for 4 hrs. **(B)** Variation of mRNA levels in response to 5-FU. In response to 4 hrs of 10 µM 5-FU, levels of the 8 mRNAs of interest that were purified from cytosolic lysates of HCT-116 cells were quantified using RT-qPCR. While no variation in translation was observed for most of the genes of interest, some genes showed an increase in their cytosolic mRNA levels in response to 5-FU, indicating that 5-FU induces transcriptional regulation of some mRNAs at early time point. **(C)** Comparison of Translation Index (TI) and relative mRNA levels. Using the 8 genes of interest, Translational Index (TI) and relative variation in mRNA levels in response to 4 hrs of 5-FU exposure in HCT-116 cells were compared. No significant correlation was observed between TI and mRNA levels, suggesting that variations in translation do not result from variation in transcriptional events affecting the mRNA of interest. Graphs present means and SD of two independent experiments.

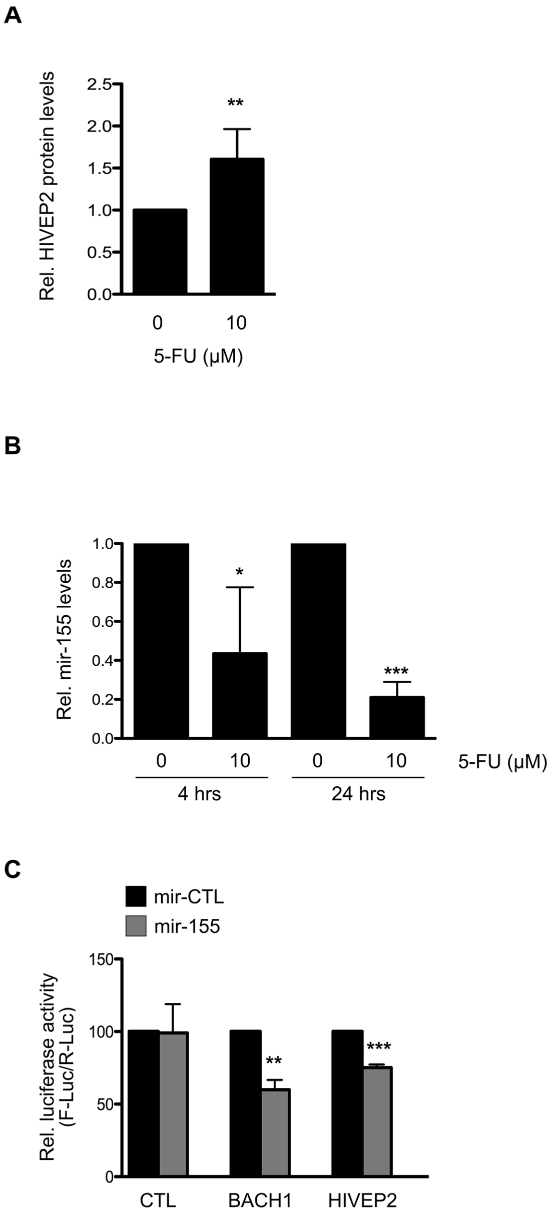

Supplementary Figure 5: Translational regulation of *HIVEP2* gene by mir-155 in HCT-116 cells. (A) Expression of HIVEP2 protein in response to 5-FU in HCT-116 cells. Quantification of HIVEP2 protein levels analysed by Western blot. 5-FU treatment increases HIVEP2 protein. **(B)** Expression of mir-155 in response to 5-FU exposure in HCT-116 cells. mir-155 expression levels in response to 10 µM of 5-FU at 4 and 24 hrs post-treatment were analysed by RT-qPCR. A significant decrease in mir-155 levels was observed from 4 hrs post-treatment, indicating that 5-FU inhibits mir-155 expression and/or processing at early time point. **(C)** Impact of mir-155 on 3'UTR activities of known target genes. Luciferase assays were performed using a reporter assays containing either no 3'UTR sequence downstream *Firefly* luciferase gene (CTL) or 3'UTR sequence of *BACH1* or *HIVEP2* genes. These systems were co-transfected in HCT-116 cells with either a plasmid expressing mir-155 or an empty vector (mir-CTL). Expression of mir-155 significantly reduced luciferase activity of both BACH1 and HIVEP2 reporter plasmids without affecting the one of CTL reporter plasmid. Graphs present means and SD of at least three independent experiments. *: $P < 0.05$; **: $P < 0.01$; ***: $P < 0.001$.

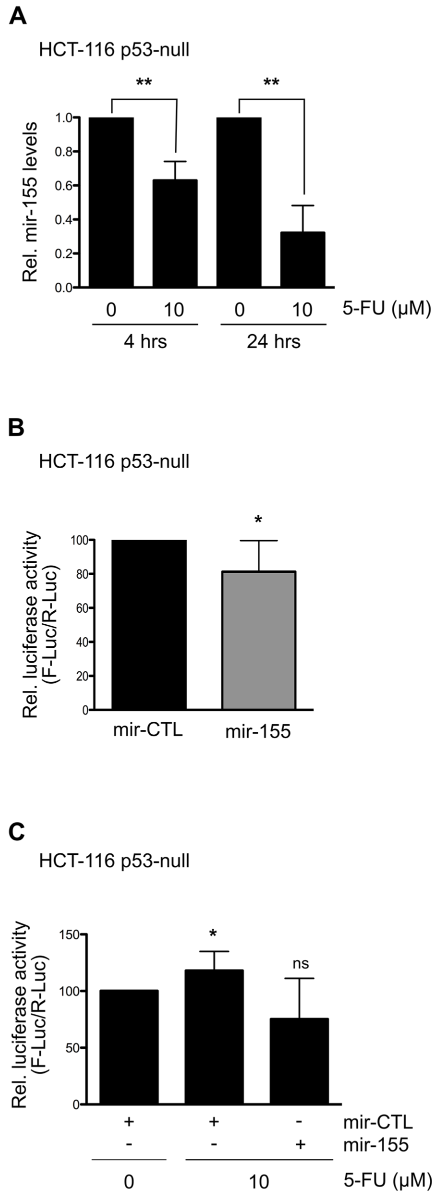

Supplementary Figure 6: Translational regulation of *HIVEP2* gene by mir-155 in HCT-116 p53-null cells. (A) Expression of mir-155 in response to 5-FU exposure in HCT-116 p53-null cells. mir-155 expression levels in response to 10 µM 5-FU at 4 hrs and 24 hrs post-treatment were quantified by RT-qPCR. Like in HCT-116 cells, a significant decrease in mir-155 levels was observed from 4 hrs post-treatment in HCT-116 p53-null, indicating that 5-FU inhibits mir-155 expression and/or processing at early time point in both p53 expressing and p53 depleted cells. **(B)** Impact of mir-155 on 3'UTR activities of *HIVEP2* gene. Luciferase assays were performed using a reporter assays containing 3'UTR sequence of *HIVEP2* gene downstream *Firefly* luciferase gene. This system was co-transfected in HCT-116 p53-null cells with either a plasmid expressing mir-155 or an empty vector (mir-CTL). Expression of mir-155 significantly reduced luciferase activity of HIVEP2 reporter plasmids. **(C)** Analysis of HIVEP2 translational regulation through its 3'UTR using luciferase reporter assays in HCT-116 p53-null cells. Luciferase activity was measured to determine the impact of HIVEP2 3'UTR on translation in response to 0 or 10 µM 5-FU for 24 hrs in presence (mir-155) or absence (mir-CTL) of mir-155. In HCT-116 p53-null cells, over-expression of mir-155 is sufficient to abolish the increased activity of 3'UTR HIVEP2 in response to 5-FU treatment, indicating that mir-155 inhibits *HIVEP2* translation in response to 5-FU. Graphs present means and SD of at least three independent experiments. *: $P < 0.05$; **: $P < 0.01$; ***: $P < 0.001$.

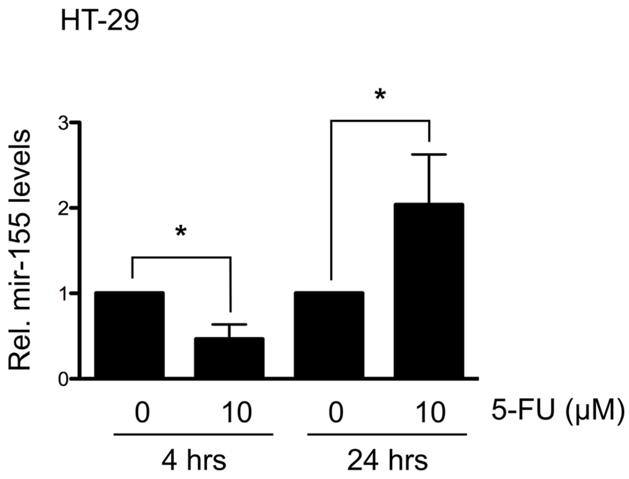

Supplementary Figure 7: Expression of mir-155 in response to 5-FU exposure in HT-29 cells. mir-155 expression levels in response to 10 µM 5-FU at 4 hrs and 24 hrs post-treatment were quantified by RT-qPCR. Like in HCT-116 cells, a significant decrease in mir-155 levels was observed from 4 hrs post-treatment in HT-29, indicating that 5-FU inhibits mir-155 expression and/or processing at early time point. In contrast to HCT-116 cells, mir-155 expression level is then significantly increased in response to 5-FU at 24 hrs post-treatment. Graphs present means and SD of at least three independent experiments. *: $P < 0.05$.

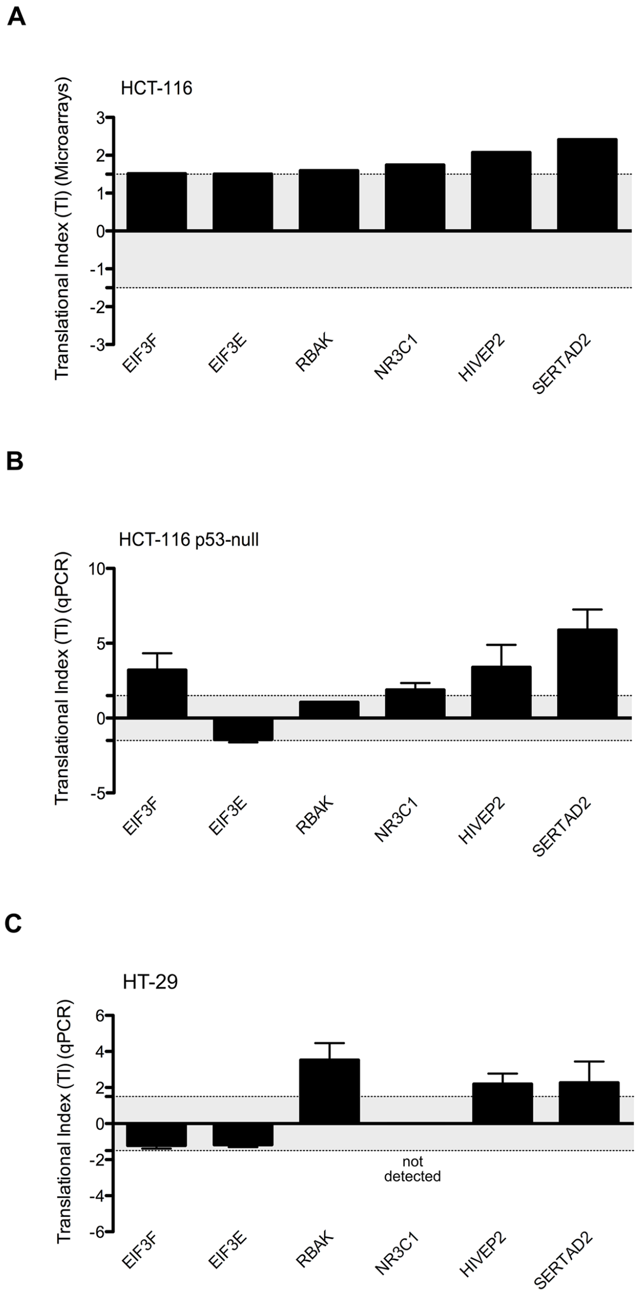

Supplementary Figure 8: Translational regulation of mir-155 target genes in response to 5-FU exposure. Translational index of 6 target genes of mir-155 were determined in response to 10 µM of 5-FU for 24 hrs in HCT-116 using microarrays **(A)**, in HCT-116 p53-null **(B)** and HT-29 **(C)** using RT-qPCR. Except for two genes out of six in HCT-116 p53-null cells and for two genes out of five in HT-29 cells, mir-155 target genes were translationnally up-regulated in the three cell lines in response to 5-FU exposure. Graphs present means and SD of at least three independent experiments.

Supplementary Table 1: Distribution of genes significantly deregulated at translational level (*P* < 0.05) in response to 10 µM 5-FU in HCT-116 cells depending on the cut-off applied.

Cut-off		1.3	1.5	2.0
**Total gene deregulated**		798	313	26
Down regulation	n (%)	167 (21%)	29 (9%)	1 (4%)
Up regulation	n (%)	631 (79%)	284 (91%)	25 (96%)

Supplementary Table 2: List of translationally deregulated genes in response to 10 µM 5-FU in HCT-116 cells (*P* < 0.05 and minimum cut-off = 1.3).

See Supplementary File 1

Supplementary Table 3: Gene ontology analysis of list of translationally deregulated genes in response to 10 µM 5-FU in HCT-116 cells using the three data sets.

See Supplementary File 2

Supplementary Table 4: Enrichment of mir-155 target genes in translationally up-regulated genes in response to 10 µM 5-FU in HCT-116 cells.

	All gene detected in microarrays	Cut off 1.5		
		All	Up	Down
Number of genes	14,727	313	284	29
Number of mir-155 target genes (%)	642 (4.4%)	19 (6.1%)	19 (6.7%)	0 (0.0%)
Number of other genes (%)	14,085 (95.6%)	294 (93.9%)	265 (93.3%)	29 (100%)
P-value[#]		0.10716	**0.0389***	0.3088

#Two-tailed *P*-value of Chi-square test (df=1)

Supplementary Table 5: Gene ontology analysis of list of translationally deregulated genes in response to 10 µM 5-FU in HCT-116 cells that are mir-155 target genes (*P* < 0.05 and cut-off = 1.5, n = 19).

Category	Term	Gene number	% gene	Fold Enrichment	p-value	Log (p-value)	Genes
**Cluster 1 – Enrichment Score: 1.533**							
GOTERM_BP_FAT	GO:0006355-regulation of transcription, DNA-dependent	6	31.579	3.270	0.020	-1.701	RBAK, MECP2, HIVEP2, NR3C1, ZKSCAN5, SERTAD2
GOTERM_BP_FAT	GO:0051252-regulation of RNA metabolic process	6	31.579	3.198	0.022	-1.661	RBAK, MECP2, HIVEP2, NR3C1, ZKSCAN5, SERTAD2
SP_PIR_KEYWORDS	transcription regulation	6	31.579	2.998	0.034	-1.464	RBAK, MECP2, HIVEP2, NR3C1, ZKSCAN5, SERTAD2
SP_PIR_KEYWORDS	transcription	6	31.579	2.933	0.037	-1.428	RBAK, MECP2, HIVEP2, NR3C1, ZKSCAN5, SERTAD2
GOTERM_BP_FAT	GO:0006350-transcription	6	31.579	2.760	0.039	-1.409	RBAK, MECP2, HIVEP2, NR3C1, ZKSCAN5, SERTAD2

Supplementary Table 6: Sequences of oligonucleotides used in the study.

Gene symbol	Primer	Sequence 5' → 3'
**ASF1B**	F	CGGTTCGAGATCAGCTTCG
	R	CAAATTCCTCACTCTCAGCC
**CHMP5**	F	CCAACAGTCATTCAACATGG
	R	TCTGGTCGATCTTCACTTGC
**EIF3E**	F	CGTCGGCAGGTTCTAAAAGA
	R	GAGCCCCATCAAAGTCAAAG
**EIF3F**	F	CACTCAGTGGAGGTCACCAA
	R	GCAAATTCCATGTCAACAGC
**FRK**	F	GTGAGCCACTACACCAAGAC
	R	GGAGTTGCGGTCTATCTCCC
**GAPDH**	F	AGCCACATCGCTCAGACAC
	R	GCCCAATACGACCAAATCC
**HIVEP2**	F	GGGAGTCTCAATGACATCGG
	R	CATCTGATTCCTCAGCATCG
**KLF4**	F	ACCCACACAGGTGAGAAACC
	F	CCCCGTGTGTTTACGGTAGT
**IL18**	F	CTGCAGTCTACACAGCTTCG
	R	GTCTTCTACTGGTTCAGCAG
**L3MBTL3**	F	GACACAGATGATCACCGGG
	R	TGAACACCAGCCTACAGGG
**LMNB1**	F	TTGTCAGAGCCTTACTGAGG
	R	CTTATACAGCCTCACTTGGG
**NR3C1**	F	ATGCCGCTATCGAAAATGTC
	R	TAGTGGCCTGCTGAATTCCT
**RBAK**	F	TGGGAGGTGAATTTCCATGT
	R	AGCAGCTTGCCTTGAATGTT
**RPL13**	F	GGTGGCCAGTTTCAGTTCTT
	R	AACAAGTCCACGGAGTCCC
**SERTAD2**	F	CCTCCTGATGCGTTAGTTCC
	R	ACAGGGAGACACGATTTTGC
**SRP68**	F	AAAGCCGTGAAGCATGCAG
	R	GAGAGGTAAGCTGTGTAAGC
**STIP1**	F	AGAGCAGCTACGAAACAAGC
	R	GTGTTGCAATCTCTTCCTCC
**TAF1B**	F	AAGGCTGGGATTGGTATGTG
	R	TGCTCTTCTGAAGGTAGCGC
**TBCD**	F	TTTCCTGAGTACACGCAGC
	R	AGCCAACTCTCGGATGACC
